# Supplementary material for: Complexome profiling of the Chlamydomonas psb28 mutant reveals TEF5 as an early PSII assembly factor
Source: Plant Cell. 2025 Mar 17;37(6):koaf055. doi: 10.1093/plcell/koaf055 (PMC12164590; doi:10.1093/plcell/koaf055)
Supplement: koaf055_Supplementary_Data [file koaf055_supplementary_data.zip › psb28_interactive.zip!/plots/intensity/qualityPlots/peptides/FTT1_Cre12.g559250.t1.2.html]

### Cre12.g559250.t1.2

Trivial name: FTT1  
SeqCoverage [median]: 37.50  
Mass: 29.51 kDa  
  
 

| Sequence | MissedCleavage | Mass [Da] | wtIntensity | muIntensity | Score |
| --- | --- | --- | --- | --- | --- |
| AAQDIALVDLPPTHPIR | 0 | 1826.01 | 799.53 | 1734.73 | 66.29 |
| EAAEHTLLAYK | 0 | 1244.64 | 4732.39 | 2964.59 | 73.63 |
| IISSIEQKEEAK | 1 | 1373.74 | 1485.04 | 3475.92 | 63.66 |
| IISSIEQKEEAKGNEEHVQR | 2 | 2323.18 | 0.00 | 1818.96 | 110.44 |
| LAEQAER | 0 | 815.41 | 374.76 | 1757.21 | 142.36 |
| LAEQAERYDEMVEEMKK | 2 | 2097.97 | 0.00 | 1225.20 | 98.51 |
| LVHDQELSVEER | 0 | 1452.72 | 12448.15 | 14360.19 | 113.24 |
| NLLSVAYK | 0 | 906.52 | 4725.05 | 5431.02 | 113.70 |
| YLAEFK | 0 | 769.40 | 2544.07 | 2535.34 | 106.29 |
|  |  |  | 27109.0 | 35303.2 |  |
